# Evaluating the Accuracy and User Satisfaction of Culturally-Relevant Kannada-Based Hearing Screening App Compared to Technical Screening Method

**DOI:** 10.1055/s-0046-1817134

**Published:** 2026-05-05

**Authors:** Hemanth Narayan Shetty, Juditha Tina

**Affiliations:** 1 Department of Audiology, JSS Institute of Speech and Hearing, Mysuru, Karnataka, India; 2 Postgraduate program JSS Institute of Speech and Hearing, Mysuru, Karnataka, India

**Keywords:** sensitivity, specificity, threshold, accuracy, screening, detection

## Abstract

**Introduction:**

Non-technical hearing screening using a self-assessment questionnaire in a mobile app offers a low-cost solution for at-risk populations. The present study developed Kannada-language hearing questions using health literacy principles—plain language, simple phrasing, and dialect inclusion—to improve accessibility for underserved groups.

**Objective:**

To evaluate the effectiveness of culturally relevant Kannada hearing questions in detecting hearing loss compared with technical screening. The objectives were to assess the content validity of the new questionnaire, compare the accuracy of technical and non-technical screening apps against conventional pure-tone audiometry, and measure client satisfaction.

**Study Sample and Design:**

Eighty-four participants aged 20 to 45 years with minimal-to- moderate hearing loss were evaluated using a comparative research design.

**Methods:**

A standardised adult hearing questionnaire was developed and content-validated. Each participant underwent hearing screening with both technical and non-technical mobile apps, followed by a client satisfaction survey. Conventional pure-tone audiometry (0.5–8 kHz) was used to determine hearing thresholds.

**Results:**

The technical screening app demonstrated 97% sensitivity and accuracy, with 95% specificity. Although the non-technical app initially showed lower accuracy, employing a two-refer threshold improved its performance to 86% sensitivity, 100% specificity, and 97% accuracy. Additionally, user satisfaction ratings were higher for the non-technical app.

**Conclusion:**

With a two-refer threshold, the accuracy of the Kannada non-technical screening app was comparable to that of the technical screening method.

**Clinical Relevance:**

The self-guided technical app benefits individuals with technical proficiency, while the non-technical screening is ideal for native speakers with dexterity issues or limited technical skills.

## Introduction

The global prevalence of hearing loss is a significant public health concern. An estimated 460 million people have disabled hearing impairment globally. 1 Due to population growth and aging, projections indicate that by 2025, over 700 million people will have disabling hearing loss, increasing with age and affecting over 25% of individuals older than 60. 2 Hearing loss in India affects 6.6% of 109 crores, with a higher prevalence in rural areas. 3 Many lower-income or middle-income groups experience a significant lack of hearing healthcare professionals, resulting in hearing loss often going unidentified and impairment being unmanaged. 4 Early hearing screening among the broader population helps in the early detection of hearing loss, which consumes less time and is cost-effective. 5 6 We can find more hearing screening applications in the Play Store of Android (Google LLC) and the App Store of the iPhone (Apple Inc.) operating systems. 7 A few hearing screening applications are technically driven, and a few are not.

Technical hearing screening is an application installed on personal mobile phones to track their hearing threshold using the adjustment method. Given their low cost and accessibility, technical hearing screening applications may facilitate screening as a good initiative approach for assessing hearing ability. 8 Hearing screening applications show better acceptance among users because of their simplified procedure in assessing hearing ability, requiring no assistance during testing, navigation from one page to another through self-instruction features, and applications loaded into their devices can be administered at their convenience. 5 Furthermore, smartphone-based technical hearing screening may be an accurate and accessible approach to hearing evaluations, especially in settings where conventional pure tone audiometry (PTA) is unavailable and there is limited access to hearing care. 9 10 Furthermore, check their hearing ability before getting confirmation from an audiologist. The smartphone based hearing screening tool demonstrated a sensitivity of 91% for mild hearing loss and 94% for moderate hearing loss, along with the specificity of 90%, These findings support its suitability for doorstep hearing screening and large scale epidemiological assesments. 11 The functionality of application interfaces, 12 background noise levels, 13 the kind of headphones used, and their calibration, as well as individual skill levels and dexterity, 14 can significantly influence the success of hearing screenings. Tackling these issues can improve the dependability and precision of hearing assessments conducted through apps.

Non-technical hearing screening is the standardized self-assessment hearing-related questionnaire integrated into a mobile application. Considering the vast population at risk for hearing loss, a questionnaire-based assessment tool works with low operating costs. 15 Framing new questions in non-technical hearing screening should ensure that cultural and contextual relevance influence the effectiveness and relevance of the screening tools. Thus, in the present study, the hearing-related questions were developed in the Kannada language, considering health literacy principles such as plain language, short and straightforward questions, local dialects, and regional linguistic variations so that people can easily understand the questions. Framing questionnaire-based assessment tools demands no expensive equipment, especially in developing countries with low per capita income. 16 It helps individuals self-assess their hearing in less time 17 and requires no skill sets, unlike smartphone hearing screening applications. The sensitivity and specificity of non-technical hearing screening applications were 70% and 72%, respectively. 18 It is speculated that more ‘refer’ in the standardized questionnaire may increase sensitivity.

It is important to obtain client satisfaction for application quality checks. The client satisfaction questionnaire measures satisfaction with services received by individuals, while highlighting the scope for improvement 19 in the revised version of the application or when developing a new application with a similar function. The client satisfaction questionnaire provides efficient, sensitive, and reasonably comprehensive measures of patient/client satisfaction with services received.

The purpose of the study aligns with the overall goal of determining how effectively the newly developed culturally and contextually relevant questions related to hearing in the Kannada language can detect the presence and absence of hearing loss compared with the technical screening method. It is also crucial to evaluate how users experience the screening tools regarding usability, accessibility, and perceived reliability since these elements can influence their implementation in widespread hearing screening initiatives. The following objectives were formulated: a) to determine the content validity of the newly developed non-technical hearing screening questionnaire; (b) to compare hearing thresholds at each frequency between conventional audiometry (CA) and technical hearing screening app; (c) to compare screening results from both app types against CA stratified by degree of hearing level; (d) to determine the sensitivity, specificity, and predictive values of both app types and (d) to assess client satisfaction between the two apps.

## Methods

A comparative research design was used to assess the accuracy of technical and non-technical hearing screening apps in detecting the presence and absence of hearing loss.

### Participants

A convenient and purposive sampling method was employed in the current study. Patients who came to the Audiology Department for a hearing assessment were administered non-technical and technical hearing tests during their waiting period. A total of 84 native speakers of Kannada, aged 20 to 45 (mean = 32 ± 8.2) years, were included in the study. The sample size was based on an effect size of 0.41 from a preliminary study of Hazan et al. 20 regarding hearing thresholds at 500 Hz, comparing CA with the DuoTone app. Using G*Power, with a significance level of 0.05 and a power of 0.95, we determined that 64 participants per group were needed. To account for a 30% participants who might refuse to give consent, we increased the sample size by 20, resulting in a total of 84 participants planned. The age range of 20 to 45 years was intentionally chosen to evaluate the usability of both screening applications. This decision was made to reduce confounding factors, such as cognitive or motor decline related to aging, which could affect app navigation or comprehension. Recruited participants who had hearing loss ranging from minimal to moderate were included, as there is a likelihood of false-positive and false-negative responses. 21 Education level influences digital literacy and app navigation. Thus, 78% of participants recruited had completed a degree, while the rest had completed a preuniversity course. Participants with severe-to-profound hearing loss were excluded because their hearing loss is evident, and self-assessment apps may not be suitable for those with profound impairment due to floor effects. Participants with middle ear infections and psychological, neurological, and systemic illnesses were excluded from the study. Informed consent was taken from the study participants. The JSS Institute of Speech and Hearing ethics committee has approved the conduct of the study. Hearing threshold from technical hearing applications was obtained in a relatively noise-free listening environment. Standardized conventional PTA was conducted in a sound-treated room, ensuring that the measured noise level remained within the permissible range according to American National Standards Institute (ANSI) standards.

### Pure-tone Audiometry

A dual-channel diagnostic audiometer (Piano Inventis, Piano Plus Inventis), with calibrated TDH 39 Telephonics Corporation, was used to determine air and bone conduction thresholds. Hearing sensitivity thresholds for each participant were measured bilaterally across the frequency range of 0.25 to 8 kHz (in octaves) in air conduction mode. A modified Hughson-Westlake procedure measured the participant's hearing thresholds with intensity increments of +5 dB for ‘no response’ and decrements of -10 dB for ‘response.’ The pure tone average was assessed and categorised based on the severity of hearing loss. This categorization enabled a comprehensive comparison between the outcomes of technical hearing applications and those obtained from standard PTA. This analysis improves our understanding of the effectiveness of the technical screening method compared with the conventional standard method for various degrees of hearing impairment.

The technical hearing application is the Hearing Test e-audiologia.pl (Radwanice), version 2.4, which was downloaded from the Google Play store and installed on designated test mobile devices. Detailed instructions were provided to participants, guiding them to track their hearing thresholds in a quiet listening environment. Each participant's hearing threshold in air conduction mode was determined using the adjustment threshold in the frequency range of 0.25 to 8 kHz (in octaves). The ambient noise level was measured using a sound level meter (B & K 2238, BRÜEL & KJÆR company) during the test to ensure the accuracy and reliability of the testing process. If the ambient noise exceeded a predefined threshold of 35 dBA, testing procedures were temporarily paused until the noise level decreased to acceptable limits. This proactive preventive strategy enhances the precision of the outcomes.

During the assessment, participants' hearing thresholds were determined as the minimum intensity level at which they could reliably detect the tone 50% of the time. Thresholds above 15 dB HL were marked as ‘refer’, suggesting potential hearing loss, while those below 15 dB HL were marked as ‘pass,’ indicating normal hearing. This classification helped to assess participants' hearing status and guided recommendations for more detailed evaluations.

### Development of a Hearing-related Questionnaire

Ten speech and hearing professionals, each with a minimum of 5 years of experience, were involved in the content validation of the questionnaire. During the item generation process, the most commonly reported symptoms among adults from the literature review were used to create the questions. A total of seven questions were developed. The investigators carefully examined the questionnaire items for clarity, relevance, content load, and potential ambiguity. The questionnaire comprised dichotomous, closed-ended questions with ‘yes’ or ‘no’ response options. It was emailed to the experts with the necessary information and instructions for the validation process. The experts were asked to rate the questionnaire items on a five-point scale, according to which five indicated the highest appropriateness level and one indicated the lowest. They were also encouraged to provide descriptive feedback on the items. Ten days were given for the experts to complete the validation. After content validation, the questions were integrated into the mobile app.

### Non-technical Hearing Screening Application

Participants were instructed to self-administer or take the help of informants who could read the questions from the mobile application. Seven questions were presented sequentially to each participant to evaluate their hearing ability and auditory experiences. Participants answered questions about speech comprehension in noisy and quiet environments, tinnitus presence, and any feelings of blockage or irritation in their ears. They responded with a simple ‘yes’ or ‘no’. A ‘yes’ response was marked as ‘refer,’ indicating a potential hearing issue that needed further assessment. This approach effectively identified even subtle signs of hearing challenges or discomfort.

### Client Satisfaction Questionnaire

After each hearing screening test, participants answered three questions to evaluate their satisfaction with the experience. These questions were thoughtfully developed to delve into various aspects of their interaction with the non-technical or technical hearing screening application and assess their likelihood of recommending it to others, along with their inclination to reuse it soon.

1. “Did the non-technical/technical hearing screening application meet your need to assess hearing sensitivity?”
2. “How likely were you to recommend a family member or friend to use this app?”
3. “How likely were you to use this app again?”

The first question probed participants' perception of the app's effectiveness in addressing their requirements to assess hearing sensitivity. The study aimed to assess whether the app effectively evaluated its auditory capabilities. Participants reflected on their experiences to gauge how well the app met their expectations, offering valuable feedback on its functionality in aiding their hearing assessments.

The second question delved into participants' willingness to endorse the app to their family members or friends. By asking participants to assess their likelihood of recommending the app to their close acquaintances, this question aimed to gauge their confidence level in its efficacy and reliability. Participants discussed the app's value and potential impact on their lives and social circles, emphasizing its acceptability and usefulness.

Lastly, the participants were asked to evaluate the app's future use by indicating their likelihood of using it again. This question aimed to assess their satisfaction with the app's user experience and its potential as a tool for managing hearing health. The responses highlighted areas for improvement to enhance user engagement and satisfaction.

### Statistical Analysis

A paired sample *t* -test was used to compare the hearing thresholds obtained from standard CA and technical hearing screening applications at each frequency. The Bland-Altman plot was applied to determine agreement in hearing thresholds obtained in CA and technical hearing screening applications. Sensitivity and specificity assessed the accurate identification of true positives and negatives, while positive predictive value (PPV) and negative predictive value (NPV) reflected the screening's accuracy in detecting hearing loss. Furthermore, the receiving operating curve (ROC) offered diagnostic significant values. The paired sample *t* -test assessed client satisfaction between the non-technical and technical hearing screening applications.

## Results

### Content Validation

The item content validity index (I-CVI) was calculated, as reported in Yusoff et al. 22 The ICVI for the 7 questions ranged from 0.84 to 1. Furthermore, the Kappa coefficient was performed on binary values received from 10 experts on each question, and the values ranged from 0.9 to 1. Thus, all seven questions were considered.

### Hearing Threshold between Conventional Audiometry and a Technical Hearing Screening Application

**Fig. 1:**
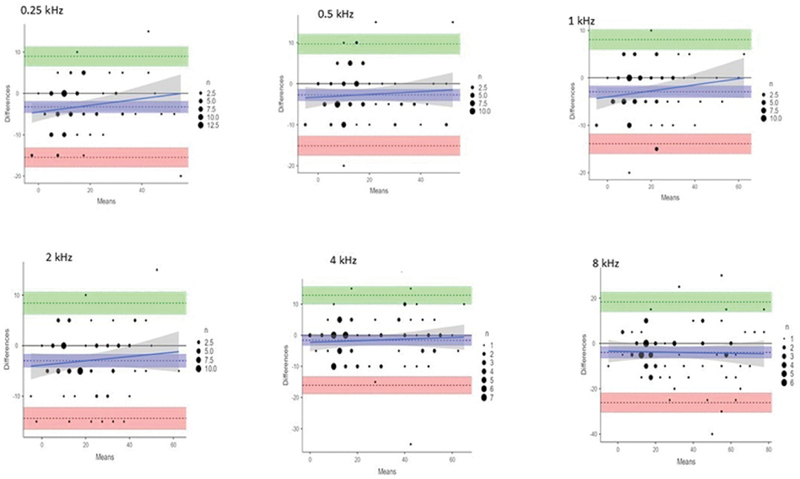
Bland-Altman plots comparing audiometric results for conventional audiometric testing versus the technical hearing screening application. There is an agreement on the threshold between conventional audiometric testing and technical hearing screening applications at each of the frequencies.

A paired sample *t* -test was performed, and the results revealed a significantly lower threshold in each frequency (0.25–8 kHz in octaves) obtained through CA than those obtained through technical hearing screening applications ( Table 1 ). Fig. 1 represents the Bland-Altman plots showing strong agreement in hearing threshold between technical hearing screening applications and CA. The average difference line lies at or close to zero at each frequency.

**Table 1:** Comparison of hearing thresholds between technical hearing screening application and conventional audiometry (N = 84; df = 83)

Frequency	Technical hearing screening application				Conventional audiometry			
	Mean	SD	*t*	*p*	Mean	SD	*t*	*p*
250 Hz	13.63	12.94	-4.81	0.001 ^a^	16.90	11.99	-4.81	0.001 ^a^
500 Hz	14.52	13.29	-3.96	0.001 ^a^	17.26	12.88	-3.96	0.001 ^a^
1,000 Hz	15.89	14.14	-4.76	0.001 ^a^	18.80	13.29	-4.76	0.001 ^a^
2,000 Hz	18.86	14.61	-4.69	0.001 ^a^	21.84	14.03	-4.69	0.001 ^a^
4,000 Hz	24.46	17.83	-1.99	0.049 ^a^	26.07	17.40	-1.99	0.049 ^a^
8,000 Hz	29.64	22.00	-3.17	0.002 ^a^	33.57	22.30	-3.17	0.002 ^a^

**Abbreviations:** df, degrees of freedom; SD, standard deviation.

**Note:** Standard deviation.

### Sensitivity and Specificity of Technical Hearing Screening Application vs Standard Conventional Audiometric Test

The pass rate was lower (-2.4%), and the referral rate was higher (2%) for the technical hearing screening application about CA ( Table 2 ). Furthermore, the detection of hearing loss among participants with stratified hearing loss was compared between technical hearing screening applications and CA. The technical hearing screening method slightly elevated participants' normal hearing thresholds to a mild degree of hearing loss, leading to a 2.3% false positive rate compared with standard CA. However, results for minimal and moderate degrees of hearing loss were consistent between the technical hearing screening and conventional methods ( Table 3 ). In addition, the Spearman's rho correlation coefficient revealed a significantly strong positive correlation in the degree of hearing loss between the technical hearing screening application and CA ( *N* = 84, r = 0.951, *p* < 0.001). The correlation coefficient suggests a high degree of consensus between the two tests in assessing the degree of hearing impairment.

**Table 2:** Comparison of pass and refer results between technical hearing screening application and conventional audiometry (N = 84)

	Technical hearing screening application		Conventional audiometry	
	Frequency	Percentage	Frequency	Percentage
Pass	45	53.6	47	56.0
Refer	39	46.4	37	44.0

**Table 3:** Comparison of the degree of hearing loss between technical hearing screening application and conventional audiometry (N = 37)

	Technical hearing screening application		Conventional audiometry	
	Frequency	Percentage	Frequency	Percentage
Minimal	14	16.7	14	16.7
Mild	17	20.2	15	17.9
Moderate	8	9.5	8	9.5

Table 4 shows that, in relation to CA, the technical hearing screening application exhibited a sensitivity of 100%, indicating its ability to identify individuals with hearing impairment correctly. Additionally, it showed a specificity of 96%, which signifies its ability to identify individuals with normal hearing correctly. The analysis of the NPV of the technical hearing screening application exhibited a 100% negative test result. At the same time, PPV values of technical hearing screening applications revealed that 95% of the test results were positive. The overall technical hearing screening application accuracy was 97%, which means that the test result correctly identified 97% of the participants having normal hearing as ‘pass’ and hearing loss as ‘refer.’

**Table 4:** Sensitivity and Specificity analysis - degree of hearing loss between technical hearing screening application and conventional audiometry

	TP	FP	FN	TN	Sensitivity (TP/ (TP + FN)	Specificity (TN/ (TN + FP)	PPV (TP/ (TP + FP)	NPV (TN/ (TN + FN)	Accuracy (TP + TN/ Total)
THE versus CA	37	2	0	45	1	0.96	0.95	1	0.97
Minimal HL	14	0	0	45	1.00	1.00	1.00	1.00	1
Mild HL	15	2	0	45	1.00	0.96	0.88	1.00	0.96
Moderate HL	8	0	0	45	1.00	1.00	1.00	1.00	1

**Abbreviations:** CA, conventional audiometry; FN, false negative; FP, false positive; NPV, negative predictive value; PPV, positive predictive value; THSA, technical hearing screening application; TN, true negative; TP, true positive.

In stratified hearing loss conditions, the technical hearing screening application has a sensitivity of 100% for identifying individuals with minimal and moderate hearing loss and a specificity of 100%. However, the sensitivity of identifying mild hearing loss was 100%, and the specificity was 96%. Thus, the PPV and NPV are 88% and 100%, respectively. The PPV of 88% means that the participants who were referred by the test truly have hearing loss (true positives), while 12% might not have it (false positives). Further, an NPV of 100% means that the test identifies a person as not having hearing loss (a negative result); there is a 100% chance that the person truly does not have hearing loss.

In addition, the technical hearing screening application was 100% accurate in identifying minimal and moderate hearing loss. However, the application's accuracy in identifying mild hearing loss was determined to be 96%, which means that the test result correctly identified 96% of the participants with normal hearing and mild hearing loss.

### Receiver Operating Characteristic Curve Analysis for a Technical Hearing Screening Application

**Fig. 2:**
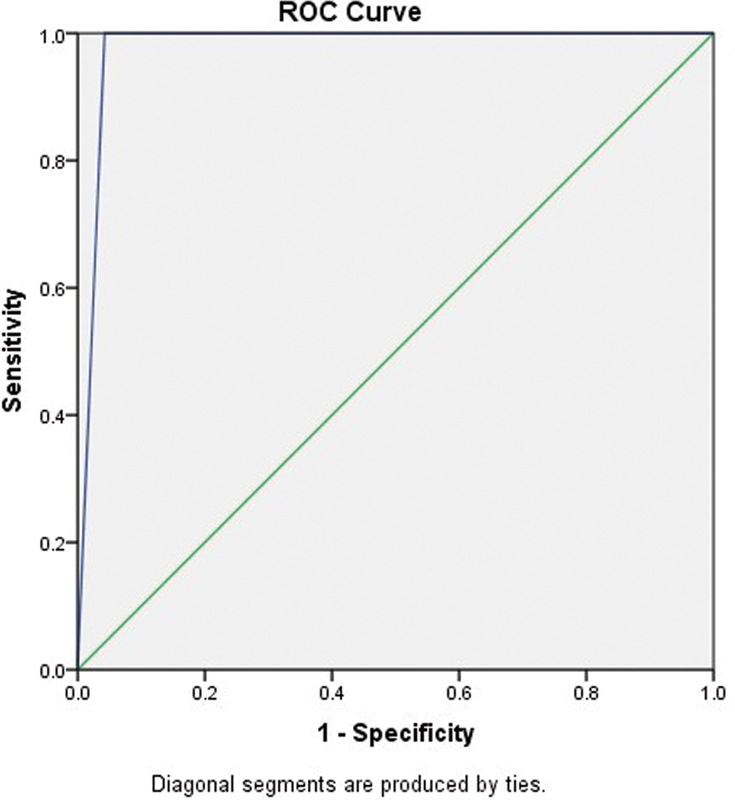
Receiver operating characteristic curve of technical hearing screening application.

The ROC analysis for the technical hearing screening application reveals an area under the curve (AUC) value of 0.97 ( Fig. 2 ). The technical hearing screening application identifies individuals with and without hearing loss accurately 97% of the time. With a standard deviation of 0.01, the AUC estimate demonstrates high accuracy and consistent performance across different samples. The confidence interval ranges from 0.94 to 1.00, with the lower limit of 0.94 indicating strong performance even at its lowest estimate. The upper bound of 1.00 suggests that the test could perform perfectly.

### Sensitivity and Specificity of Non-Technical Hearing Screening Application vs Standard Conventional Audiometric Test

The pass rate for the non-technical hearing screening application decreased by 13.1%, while the refer rate increased by 13.1% compared with CA ( Table 5 ).

**Table 5:** Comparison of pass and refer results between non-technical hearing screening application and conventional audiometry (N = 84)

	Non-technical hearing screening application		Conventional audiometry	
	Frequency	Percentage	Frequency	Percentage
Pass	36	42.9	47	56.0
Refer	48	57.1	37	44.0

Table 6 shows that the non-technical hearing screening application exhibited a sensitivity of 97%, indicating its ability to identify individuals with hearing impairment correctly. Additionally, it showed a specificity of 74%, which signifies its ability to identify individuals with normal hearing correctly. The analysis of the NPV of non-technical hearing screening applications reveals that 97% of test results are negative. It indicates a 97% chance that the individuals truly have normal hearing. In contrast, PPV values of non-technical hearing screening applications revealed that 75% of test results were positive. It implies a 75% chance that the individuals have hearing loss. The accuracy of the non-technical hearing screening application was found to be 85%. It implies that the test result correctly identified that 84% of the participants had normal hearing and hearing loss. Furthermore, the non-technical hearing screening application results were analyzed based on the number of ‘refer’ outcomes. The result ‘one-refer’ has a sensitivity of 95% for identifying individuals with hearing loss and a specificity of 81%. Thus, the PPV and NPV are 69% and 97%, respectively. However, the sensitivity with ‘two refers’ was 92%, and the specificity was 90%. In contrast, the PPV and NPV were 75% and 97%, respectively. Finally, the sensitivity of the result ‘> 2 refers’ was 86%, and the specificity was 100%. Hence, the PPV and NPV were 100% and 97%, respectively. As the number of referrals increases, the sensitivity for detecting hearing loss decreases, while the specificity for correctly identifying individuals without hearing loss improves. Additionally, the PPV rises, meaning a higher percentage of those referred by the screening have hearing loss. The NPV consistently remains at 97%, indicating that 97% of participants who pass the screening genuinely do not have hearing loss, regardless of the number of referrals.

**Table 6:** Sensitivity and specificity analysis between non-technical hearing screening application and conventional audiometry

	TP	FP	FN	TN	Sensitivity (TP/ (TP + FN)	Specificity (TN/ (TN + FP)	PPV (TP/ (TP + FP)	NPV (TN/ (TN + FN)	Accuracy (TP + TN/ Total)
**NTHSA** **versus Conventional**	36	12	1	35	0.97	0.74	0.75	0.97	0.85
**NTHSA 1R Vs Conventional**	18	8	1	35	0.95	0.81	0.69	0.97	0.85
**NTHSA 2R Vs Conventional**	12	4	1	35	0.92	0.90	0.75	0.97	0.90
**NTHSA > 2R Vs Conventional**	6	0	1	35	0.86	1.00	1.00	0.97	0.97

**Abbreviations:** FN, false negative; FP, false positive; NPV, negative predictive value; NTHSA, non-technical hearing screening application; PPV, positive predictive value; TN, true negative; TP, true positive.

### Receiver Operating Characteristic Analysis for a Non-technical Hearing Screening Application

**Fig. 3:**
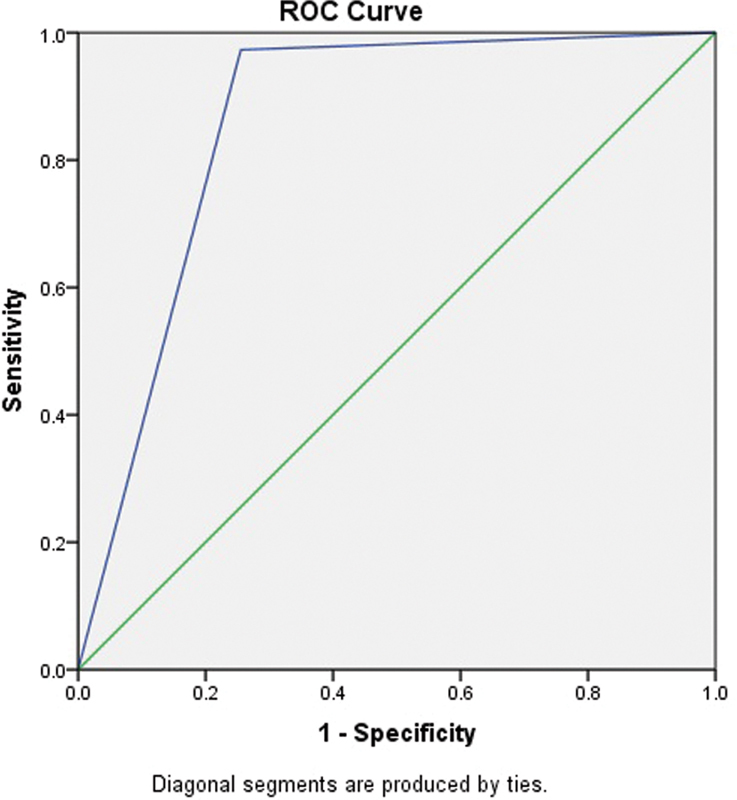
Receiver operating characteristic curve of non-technical hearing screening application.

The ROC analysis for non-technical hearing screening applications reveals an AUC value of 0.85 ( Fig. 3 ). Non-technical hearing screening tools identify individuals with and without hearing loss 85% of the time. The standard deviation of 0.04 indicates moderate precision in the AUC estimate, showing variability across samples. The confidence interval of 0.77 to 0.94 confirms reliable detection of hearing loss, even in less favorable conditions. At the same time, the upper limit of 0.94 reflects strong performance in differentiating individuals with and without hearing loss.

### Client Satisfaction Questionnaire Comparing Technical and Non-technical Hearing Screening Applications

The non-technical hearing screening application received significantly higher mean scores than the technical application across all evaluated questions: for question 1, “need of assessing hearing sensitivity” (M = 3.40, SD = 0.54 versus M = 3.16, SD = 0.69; t = -0.37, *p* = 0.001); for question 2, “need to recommend the app to family or friends” (M = 3.42, SD = 0.54 versus M = 3.23, SD = 0.61; t = -0.31, *p* = 0.003); and for question 3, “need to use the app again” (M = 3.40, SD = 0.58 versus M = 3.19, SD = 0.67; t = -0.34, *p* = 0.002).

## Discussion

The ability to accurately detect hearing loss through both technical and non-technical screening tools is crucial. It empowers individuals to make informed choices about their options. The present study evaluated the accuracy of technical and non-technical hearing screening applications compared with CA. Technical hearing screening applications showed significantly poorer hearing thresholds compared with CA. 23 The difference in threshold between hearing screening and CA ranges from 3 to 4 dB across frequencies from 250 to 8 kHz. The difference in threshold is slight, within the standard deviation of the threshold obtained either by CA or a technical hearing screening application. The expected variability of hearing threshold measurements is 3 to 4 dB.

Additionally, the application revealed significant positive correlations between CA and technical hearing screening threshold. 8 As expected, the poorer response compared with CA is because the testing was performed by tracking their threshold using the method of adjustment, contrary to CA, where a professional tracks the threshold using equipment to deliver accurate output through calibrated transducers in a sound-treated environment. 24 Thai-Van et al. 25 reported a mean difference between the audiometric test results (online digital audiometry and CA) within 5 dB HL for air and bone conduction thresholds at all tested frequencies.

The sensitivity and specificity of technical hearing screening demonstrated higher values, with a sensitivity of 100%, indicating the test results are highly reliable and correctly identify the individuals who have a hearing loss, ensuring that no cases are missed, as there were no false negatives. 26 The ROC curve analysis for technical hearing screening application reveals that the AUC value is 0.97 as it accurately distinguishes between individuals with and without hearing loss 97% of the time, and the overall accuracy of technical hearing screening application is 97%, which identifies the participants having normal hearing as ‘pass’ and hearing loss as ‘refer.’ 27 The specificity of 96% is lower because the test had a high number of false positives, which suggests that some individuals have hearing loss when they do not. 28 It could be because many different types of earbuds/headphones are readily available to the user, and not all types of transducers are calibrated. 29 Positioning of the earbud in the ear canal can also vary due to user placement. It can sometimes create sound leakage due to an improper seal of the ear canal, such that external noise enters and there is a possible chance of masking it. 30

Additionally, the presence of ambient noise in the test location may affect the threshold and consequently reduce the specificity. 31 Furthermore, technical hearing screening applications had a sensitivity of 100% and a specificity of 100% for identifying individuals with minimal and moderate hearing. However, the sensitivity of identifying mild hearing loss was 100%, and the specificity was 96%. 29 The accuracy of technical hearing screening applications in identifying minimal and moderate hearing loss was 100%. However, the accuracy of technical hearing screening applications in identifying mild hearing loss was 96%. The reason for reduced accuracy is unknown, especially when identifying mild hearing loss. 5 Overall, technical hearing screening accurately distinguished between individuals with and without hearing loss. 32 Technical hearing screening applications can be used for individuals who are more familiar with technical knowledge and have no physical dexterity to use the application. 23

The ROC curve analysis for non-technical hearing screening applications reveals that the AUC value was 0.85, as it accurately distinguished between individuals with and without hearing loss 85% of the time. 27 Furthermore, the overall accuracy of non-technical hearing screening applications was 85%, and it increased to 97% with several referrals on items of questions because the questions capture the symptoms and consequently suspect hearing loss. 33 Non-technical hearing screening had a NPV of 97%, effectively assuring those who passed that they likely did not have hearing loss, which reduces unnecessary concerns and healthcare visits. It also had a PPV of 100%, confirming that anyone identified with hearing loss has it. It makes the screening an effective tool, particularly in settings with limited resources for comprehensive audiometric testing or where initial screening is needed. This early detection can prompt timely interventions or referrals to audiologists for further evaluation, potentially mitigating the progression of hearing loss. 24

Furthermore, the assessments of user satisfaction with the hearing screening applications guide us in understanding specific client concerns to improve overall service quality and help identify areas to enhance the hearing screening application's usability, accuracy, and overall user experience. Users preferred the non-technical hearing screening application experience over the technical hearing screening application, as reflected in the patient satisfaction questionnaire. Participants were more satisfied with the non-technical than the technical hearing screening application in terms of user interface and experience. It might have been due in part to the faster and simpler testing experience, as the non-technical hearing screening application provides a similar test format, which is user-friendly with a shorter testing time. Non-technical hearing screening applications with a user-centric approach and ease of use may enhance accessibility.

In summary, individuals with technical skills can use self-instructions in technical hearing screening applications for their hearing tests. Non-technical tests are better suited for those with dexterity challenges or limited technical skills. While technical applications may provide greater accuracy and advanced features, non-technical ones are more accessible, adaptable, cost-effective, and inclusive for various populations.

## Conclusion

The accuracy of hearing screening applications is an important yardstick for referring patients for confirmatory gold-standard CA. The accuracy of technical and non-technical hearing screening applications with > 2R criteria was 97%, and they can be used based on user preference and familiarity with technology.

## Limitation and Future Indication

The sample size of the current study is small for diagnostic accuracy studies, especially for subgroup analyses by hearing loss degree, and convenient sampling may have introduced bias. Future studies with randomized sampling and larger populations are needed to validate these findings. The age range (20–45 years) limits generalizability so that broader ages should be examined in future research. Gender distribution should be analyzed in upcoming studies with larger samples.
